# Pair production tomography enables imaging of MeV-scale gamma-emitting theranostic radionuclides

**DOI:** 10.21203/rs.3.rs-9349692/v1

**Published:** 2026-05-04

**Authors:** Biswajit Das, Robin Peter, Youngho Seo

**Affiliations:** a Physics Research Laboratory, Department of Radiology and Biomedical Imaging, University of California, San Francisco, California 94143, USA; b Nuclear Science Division, Lawrence Berkeley National Laboratory, Berkeley, California 94720, USA; c Department of Radiation Oncology, University of California, San Francisco, California 94143, USA; d Molecular Biophysics and Integrated Bioimaging Division, Lawrence Berkeley National Laboratory, Berkeley, California, 94720, USA; e Department of Nuclear Engineering, University of California, Berkeley, California 94720, USA

**Keywords:** High energy γ and pair-production, pair-production tomography, targeted alpha therapy, ^212^Pb daughter imaging, PET imaging

## Abstract

High-energy gamma-emissions significantly degrade image quality when imaging theranostic cancer radiopharmaceuticals with conventional SPECT scanners, especially at low activities used in targeted alpha therapy. PET imaging offers sensitivity and energy discrimination benefits, but is conventionally limited to positron-emitting isotopes. Pair production tomography (PPT) addresses this limitation by enabling localization of high-energy *γ* rays: when a *γ* ray with energy ≥ 2*m*_*e*_*c*^2^ (1.022 MeV) undergoes pair production, it generates an *e*^+^*e*^−^ pair, and the positron subsequently annihilates, emitting two coincident 511 keV photons that can be detected for tomographic reconstruction. In this work, we present the first experimental demonstration of PPT in the context of ^212^Pb targeted alpha therapy, using 2.617 MeV *γ* rays emitted from its decay daughter ^208^Tl. ^212^Pb-filled phantoms were successfully imaged through PPT using time-of-flight clinical and non-time-of-flight preclinical PET systems, with SPECT images acquired for comparison. Monte Carlo simulations further support the experimental data and PPT mechanism. These findings establish the feasibility of PPT imaging for high-energy *γ*-emitters and motivate improvements in timing, reconstruction, and detector technologies for novel radiopharmaceutical imaging.

## 1. Introduction

Nuclear medicine imaging has become a key component of modern healthcare by enabling noninvasive assessment of physiological and molecular processes in vivo, supporting diagnosis, treatment planning, and therapy monitoring [1–5]. In addition to the tens of millions of nuclear medicine imaging procedures performed each year worldwide, the past decade has witnessed a major expansion in the clinical use of radionuclides for cancer therapy [6–9]. The fact that many of these therapeutic radiopharmaceuticals also emit imageable *γ*-ray signatures has driven renewed interest in imaging technologies optimized for the theranostic setting [10–13]. Commercial Single Photon Emission Computed Tomography (SPECT) systems remain the standard for single *γ*-ray imaging, but are optimized for energies up to a few hundred keV [14–18] and exhibit degraded image quality at higher energies owing to increased scatter and collimator penetration, particularly for theranostic radionuclides with MeV-scale emissions in their decay chains [19–21]. This issue is especially pronounced when imaging for theranostic targeted alpha therapy (TAT), where quantitative imaging remains extremely challenging due to ultra-low (~ *μ*Ci) administered activities and an already tenuous signal-to-noise ratio (SNR). On the other hand, positron emission tomography (PET) offers highly sensitive and quantitative imaging of positron emitting radionuclides [22]. Modern PET systems achieve enhanced SNR through fast timing resolution enabled by time of flight capabilities [23], and also through large axial field of view that yields high photon-sensitivity [24, 25]. As PET imaging is often performed before therapy for planning and target verification, there is a considerable interest in leveraging existing PET infrastructure to reduce the logistical burden associated with additional SPECT systems and scans. However, PET requires positron emission, which is not present in current clinical targeted alpha therapy candidates such as ^212^Pb, ^225^Ac, etc.

Pair production may provide a route for imaging therapeutic radionuclides with PET systems without direct positron emission. When a gamma ray with energy ≥ 2*m*_*e*_*c*^2^ (1.022 MeV) undergoes pair production, it generates an electron-positron (*e*^−^*e*^+^) pair; the positron subsequently annihilates, producing two coincident 511 keV photons that can be detected for tomographic reconstruction, as with PET. Internal pair production from an excited nuclear state, which is the direct emission of a *e*^−^*e*^+^ pair without an intermediate photon, has been successfully explored for PET-like imaging of ^90^Y through the 1.7 MeV excited state of ^90^Zr [26–29]. Recent simulation studies have also proposed pair production tomography for in vivo dosimetry based on external MeV-range X-ray beams for radiotherapy [30, 31]. To date, however, pair production tomography (PPT) imaging using gamma rays emitted from therapeutic radionuclide remains totally unexplored experimentally.

One potential candidate for PPT imaging is TAT radionuclide ^212^Pb. Currently, ^212^Pb is a promising alpha-emitting radionuclide, and is the focus of several clinical trials as well as many experimental radiopharmaceuticals for further development [32–36]. ^212^Pb emits a 239 keV *γ* ray and produces an alpha-emitting daughter, ^212^Bi, functioning as an in vivo generator combining highly potent targeted radiotherapy with the possibility of theranostic imaging. While essential for verification of TAT delivery to the target and assessment of off-target toxicity, imaging of ^212^Pb using 79 keV and 239 keV have been demonstrated through phantom study using SPECT/CT [37, 38]. However, as ^212^Pb does not emit *α* particles, imaging of the parent radionuclide, whether direct or surrogate, introduces uncertainty in dose localization; therefore, imaging of the daughter radionuclides is essential to explore the daughters distribution. A high energy gamma ray, 2.617 MeV, is emitted from a daughter nuclide ^208^Tl with a probability of 36% per ^212^Pb decay [39], providing a suitable signal for PPT imaging. PPT offers a fundamentally different technique for imaging high energy photon emissions that are poorly visualized with conventional medical scanners. In this context, the proposed approach addresses three challenges simultaneously: extending nuclear imaging beyond conventional energy ranges, improving sensitivity in TAT through modern PET detection and coincidence based discrimination, and enabling specific localization of the alpha emitting component of ^212^Pb.

Despite its promise, implementing PPT presents significant technical challenges. Pair production occurs less frequently than Compton scattering at this energy, and successful detection requires precise coincidence timing, higher sensitivity, detector materials with high stopping power, and robust event selection techniques to distinguish true pair production events from scattered background. Recent advances in detector technology, including high sensitivity total body PET systems such as EXPLORER [22, 25], fast timing scanners [23, 40, 41], and improved reconstruction algorithms [41–43], have significantly advanced the field and pave the way for future adoption of this imaging approach.

In this work, we explored PPT imaging of the spatial distribution of ^208^Tl, a daughter of ^212^Pb, using 2.617 MeV gamma rays. Using both clinical and preclinical phantom studies with time of flight (TOF) PET and non-TOF PET systems configured for coincidence detection, we demonstrated the first experimental tomographic imaging derived from pair production through gamma rays emitted by radionuclide. These findings establish the feasibility of pair production based imaging for high energy gamma emitters and provide a foundation for further improvements in sensitivity, timing performance, and quantitative reconstruction. Ultimately, PPT may broaden the capabilities of nuclear medicine imaging and support accurate verification of emerging radionuclide therapies.

## 2. ^212^Pb decay and principle of pair production tomography for imaging

**Figure 1:**
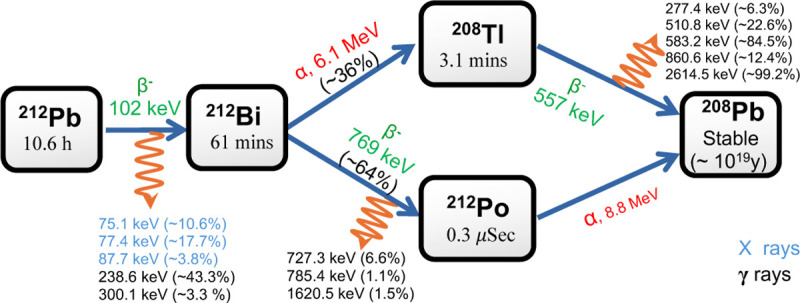
The decay chain of ^212^Pb (half life ~10.4 h) leads to stable ^208^Pb through alpha and beta emitting daughters, accompanied by multiple gamma ray emissions spanning from 75 keV to 2.7 MeV.

**Figure 2:**
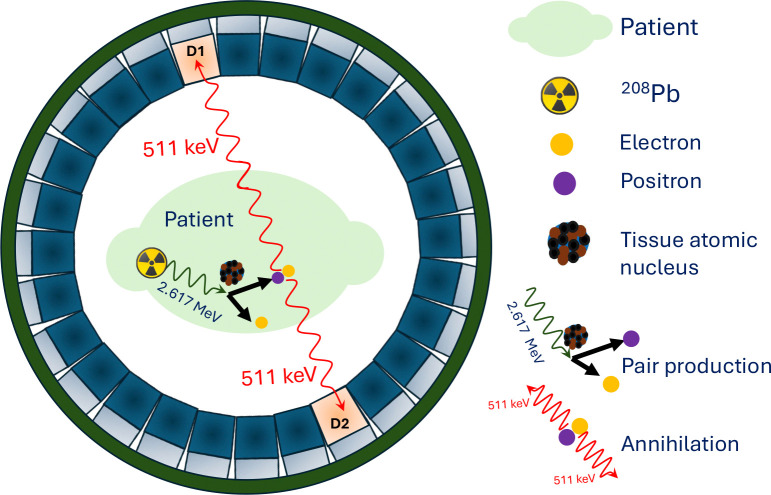
Schematic illustration of pair production (*e*^−^*e*^+^) induced by a 2.617 MeV *γ* ray, followed by electron positron annihilation producing two 511 keV photons that are detected by a pair of opposite detectors in a PET scanner for tomographic reconstruction.

In recent years, the development of novel radiopharmaceuticals based on the ^212^Pb radioisotope has advanced worldwide for theranostic applications, particularly in targeted prostate cancer therapy [44–46]. The decay chain of ^212^Pb produces gamma emissions spanning energies from approximately 75 keV–2.7 MeV, as illustrated in Fig. 1. One of its daughter radionuclides, ^208^Tl, emits a high energy gamma ray, 2.617 MeV, from an excited state populated following beta decay, with a branching ratio of ~99%. In this work, we demonstrated PPT imaging of this daughter nuclide using the 2.617 MeV gamma ray. The pair production process for this gamma ray is illustrated schematically in the inset of Fig. 2. These high energy gamma rays interact with nuclei in tissue within the patient, positioned near the center of a ring shaped PET detector array, producing an *e*^−^*e*^+^ pair. Owing to its initial kinetic energy, the *e*^+^ travels a short distance before losing energy, with an mean range of approximately 2.2 mm. This range is small compared with the intrinsic spatial resolution of PET systems. After coming to rest, the positron annihilates with an electron in the surrounding medium, producing two 511 keV photons emitted in opposite directions. Alternative annihilation channels, such as three photon emission, may also occur through ortho-positronium formation, although their branching probability is low [47–49]. The two oppositely directed 511 keV gammas can be detected in coincidence using a pair of detectors positioned on opposite sides of the PET ring, as illustrated in Fig. 2. By collecting a large number of such coincidence events, a three-dimensional distribution of the annihilation locations can be reconstructed from the detected signals. Background photon contamination arising from photoelectric absorption and Compton scattering can be effectively reduced by applying appropriate energy selection and coincidence timing window selection.

## 3. Simulation Studies

### 3.1. Simple cube phantom

The 2.617 MeV *γ* ray has a mean free path in water of 1/*μ* = 23.4 cm [50], but exponential attenuation together with isotropic 1/*r*^2^ dispersion substantially localize the source. These effects were assessed in a series of homogeneous cube phantom simulations evaluating the kinetic energy (KE) distribution, range, production distance, and annihilation distance of the positron. Monte Carlo framework GATE 9.2 (Geant4) was used to simulate *N* = 10^8^ primary 2.617 MeV *γ*-rays emitted from a central isotropic source inside the cube. To quantify *e*^+^/*e*^−^ kinetic energies and *e*^+^ ranges, full particle histories were recorded in a 10 cm × 10 cm × 10 cm water cube. For pair production and annihilation profiles, larger 20 cm × 20 cm × 20 cm cubes were simulated with four materials: water, lung tissue, adipose tissue, and bone. Details about simulation geometry, physics lists, material composition, and interpretation, are provided in the Supplementary Information.

**Figure 3:**
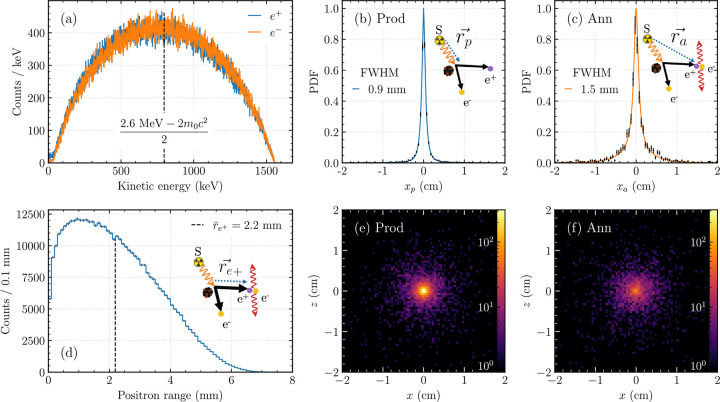
Monte Carlo simulation results: an isotropic point 2.617 MeV *γ*-ray source was placed at the center of a homogeneous cube water phantom. (a) Kinetic energy distributions of the electron (*e*^−^) and positron (*e*^+^) upon pair production. The distributions are symmetric around (2.617 MeV − 2*m*_0_*c*^2^)/2 = 0.798 MeV. (b) Slice profile of the *x*-coordinate, *x*_*p*_, of the vector from the origin to the pair production location, $\vec{r_{b}}$, with a FWHM of 0.9 mm. (c) Slice profile of the *x*-coordinate, *x*_*a*_, of the vector from the origin to the annihilation location, $\vec{r_{a}}$, with a FWHM of 1.5 mm. (d) Distribution of the positron range, $\left|\vec{r_{e+}}\right|=\left|\vec{r_{a}}-\vec{r_{p}}\right|$. The maximum positron range is approximately 7 mm, with a mean range of 2.2 mm. (e–f) X–Z center slices of the pair production and annihilation points, respectively, showing localized source signal.

Figure 3 summarizes the *γ*- and *e*^+^-range characterization of the 2.617 MeV *γ*-ray in a homogeneous water cube simulation. The KE distributions of the *e*^+^ and *e*^−^ upon pair production were roughly symmetric around (2.6 MeV − 2*m*_0_*c*^2^)/2 = 0.798 MeV (mean: 0.77 MeV), with an end-point of 1.56 MeV (Fig. 3(a)). Compared to common PET radionuclides, the *e*^+^ energy was between that of ^18^F (mean: 249.8 keV, end-point: 633.5 keV) and ^68^Ga (mean: 836.0 keV, end-point: 1.899 MeV) [39].

The position of *e*^+^/*e*^−^ production sites $\vec{r_{p}}=\left(x_{p},y_{p},z_{p}\right)$ was evaluated in a 1D slice profile of $x_{p}$ (Fig. 3(b)). The profile was normalized and fit to a Lorentzian with exponential background, $f(x)=A{\left(1+(x/\sigma {)}^{2}\right)}^{-1}+Bexp(-C|x|)$, to compute the full-width at half-maximum, ${FWHM}_{p}=0.9\;mm$. The spread of $x_{a}$ from the annihilation point $\vec{r_{a}}$ was ${FWHM}_{a}=1.5\;mm$ (Fig. 3(c)).

The mean range of individual positrons, $\left|\vec{r_{e}}\right|=\left|\vec{r_{a}}-\vec{r_{p}}\right|$, was 2.2 mm, with an end-point of approximately 7 mm (Fig. 3(d)). 2D X–Z slice images of production and annihilation events through the cube center (Figs. 3(e–f)) illustrate that despite the long range of the 2.617 MeV *γ*-ray, dispersion in a 3D medium resulted in localized signal near the point source relative to farther events, which appeared as noise.

**Figure 4:**
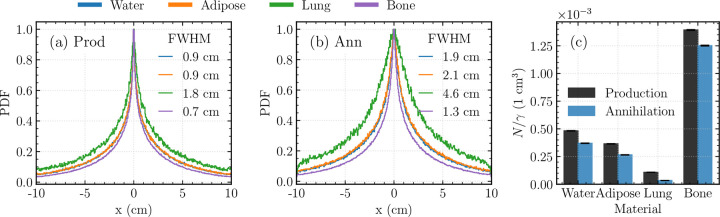
Monte Carlo study of *e*^+^ production and annihilation in homogeneous water, adipose, lung, and bone cubes (20 cm × 20 cm × 20 cm). (a) 1D x-projection profiles for *e*^+^ production in each material. (b) 1D x-projection profiles for *e*^+^ annihilation in each material. (c) *e*^+^ production and annihilation efficiencies per *γ*-emission in a (1 cm × 1 cm × 1 cm) volume centered at the origin.

Positron production and annihilation site distributions were also studied in adipose tissue, lung, and bone (Fig. 4). To obtain greater statistics, especially in low-density materials, projected *x*_*p*_ and *x*_*a*_ coordinates from the entire phantom were aggregated into 1D profiles rather than from single slices. Supplementary Fig. S1 shows how this choice increases width of a displayed 1D profile. With higher density *ρ* and atomic number *Z*, bone resulted in more localized pair-production and annihilation than materials such as lung tissue (Fig. 4(a–b)). Figure 4(c) compares the pair production efficiency between materials, defined as the number of production and annihilation events in an arbitrarily selected 1 cm × 1 cm × 1 cm cube centered about the source per primary 2.617 MeV *γ*-ray photon. As expected, bone also provided the greatest pair production efficiency, over 2.5 times relative to water and adipose tissue, and approximately 13 times relative to lung tissue. Annihilation efficiencies followed similar trends, with lung tissue exhibiting a larger decrease due to its extended annihilation range. In an infinite medium, and approximately in the full 20 cm × 20 cm × 20 cm cube (Supplementary Fig. S2), pair production and annihilation efficiencies are identical, but these data provide an additional heuristic for the signal expected to localize in a small “voxel.” Note for absolute interpretation that the ^208^Tl *γ*-ray is produced with a branching ratio of approximately 36% per parent ^212^Pb/^212^Bi decay (Fig. 1).

### 3.2. Clinical rod phantoms

**Figure 5:**
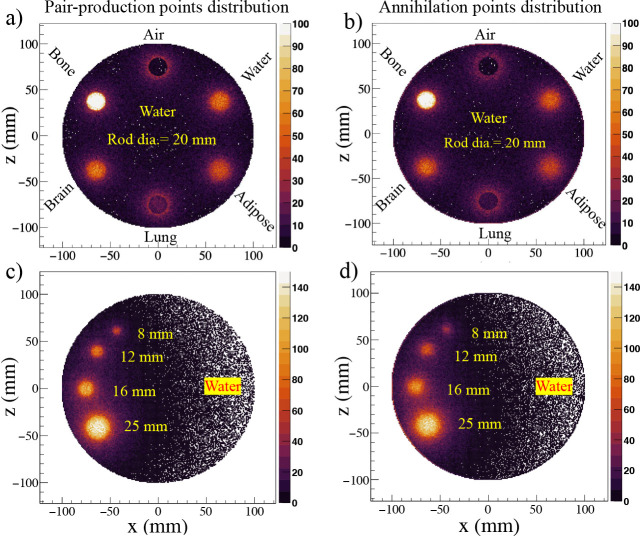
Simulation results of clinical scale cylindrical phantoms for 2.617 MeV gamma rays. (a,b) Pair production and annihilation point distributions in the X–Z plane for a multi material phantom of 2 cm diameter with six inserts (water, adipose, lung, brain, bone, and air) showing signal localization. (c,d) Pair production and annihilation point distributions of second clinical phantom with four inserts of varying diameters also showing signal localization.

We also performed simulations of two clinical scale cylindrical phantoms (20 cm diameter and 20 cm length) to investigate the distributions of pair production and annihilation points in realistic geometries. The first phantom contained six cylindrical inserts (2 cm diameter and 3.8 cm length each) composed of different materials, including water, adipose, lung, brain, bone, and air, embedded within a water-filled background. Total 10^8^ 2.617 MeV gamma rays were generated isotropically and uniformly within the volume of all inserts. The spatial distributions of pair production points and annihilation points in the X–Z plane are shown in Fig. 5(a) and Fig. 5(b), respectively. All inserts are clearly distinguishable in both the pair production and annihilation distributions. This simulation results suggest that bone, adipose tissue, and water equivalent biological samples can be imaged using PPT. However, owing to the low density of lung tissue, signal localization is limited in this region. Notably, if a cancerous tumour develops within the lung, it can be distinguished in PPT images.

The second simulated clinical phantom was designed to reflect the experimentally measured one (presented in Sec. 4.1). It consists of four cylindrical inserts with different diameters (8 mm, 12 mm, 16 mm, and 25 mm) and same length (38 mm), all filled with water with distributed source, to study the effects of insert diameter. The rest of the phantom was filled with water. A total of 10^8^ 2.6 MeV gamma rays were generated isotropically and uniformly within the volume of these inserts. The spatial distributions of pair production points and annihilation points in the X–Z plane are shown in Fig. 5(c) and Fig. 5(d), respectively. All inserts are clearly resolved down to an insert diameter of 8 mm in both the pair production and annihilation distributions, demonstrating the feasibility of PPT imaging for experimental implementation.

## 4. PPT imaging experiment and results

To demonstrate the feasibility of this new imaging concept, experiments were conducted using two different phantoms: a clinical ACR/Pro-NM PET phantom and a preclinical NU 4–2008 small animal PET phantom. Data were acquired using the Biograph Vision 600 PET/CT (Siemens Healthineers) and the NanoScan microPET/CT (Mediso) scanners for the clinical and preclinical phantoms, respectively. All experiments were conducted at the University of California, San Francisco. For the experiment, ^212^Pb was eluted in house using a ^224^Ra/^212^Pb generator developed by Pacific Northwest National Laboratory (PNNL). Controlled elution (“milking”) of ^212^Pb radionuclides was performed by passing HCl solution through the generator at a regulated flow rate.

### 4.1. Experimental demonstration with clinical phantom

**Figure 6:**
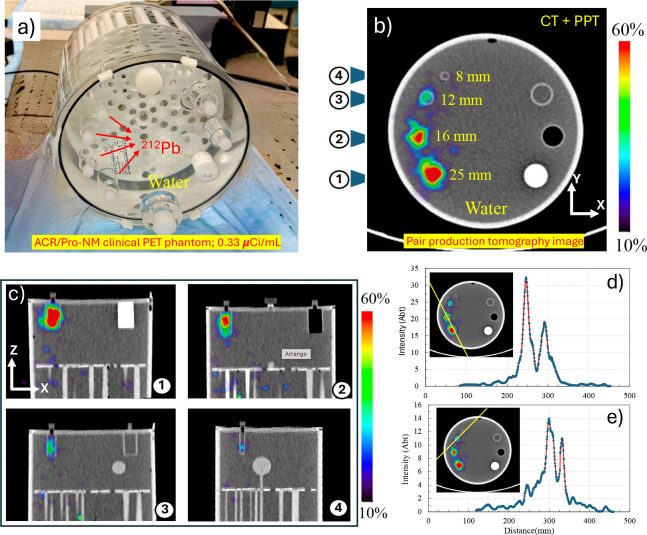
Experimental imaging results of the ACR/Pro-NM clinical PET phantom filled with ^212^Pb solution (0.33 *μ*Ci/mL) in four cylindrical inserts. a) The phantom was scanned using Biograph Vision 600 PET/CT (Siemens Healthineers) for 30 minutes. b) Pair production tomography (PPT) image reconstructed from coincident 511 keV gammas, projection on X-Y plane. c) The projection images of four inserts on X-Z plane. d-e) The line profiles of the pair production images.

We filled four cylinders (8, 12, 16, and 25 mm diameters, each 38 mm length) of the ACR/Pro-NM PET phantom with 0.33 *μ*Ci/mL of ^212^Pb (Fig. 6(a)). The background volume of the phantom was filled with nonradioactive water. The phantom was scanned using Biograph Vision 600 PET/CT (Siemens Healthineers) for 30 minutes. This PET system incorporates lutetium oxyorthosilicate (LSO) crystals with dimensions of 3.2 mm × 3.2 mm × 20 mm (l × w × t), fully coupled to silicon photomultipliers (SiPMs). Its proprietary detector architecture enables a volumetric resolution of approximately 48 mm^3^ while achieving time of flight performance of about 214 ps. Pair production tomographic reconstruction of the phantom is shown in Fig. 6(b–c) in the *X*–*Y* and *X*–*Z* planes, using coincident 511 keV events selected from opposing detectors within a coincidence time window defined by the system timing resolution. The acquired list mode data were reconstructed using the vendor-provided iterative reconstruction algorithm incorporating time-of-flight and point-spread-function deblurring as well as CT-based attenuation and scatter corrections in a 440 × 440 × 88 matrix, with 4 iterations and 5 subsets. Post-reconstruction Gaussian filter with 9 mm FWHM was applied to suppress the noise. Line profiles in two directions are also shown in Fig. 6(d–e). The reconstructed image demonstrates the feasibility of PPT imaging using a time of flight PET scanner. The three rods inserts are clearly resolved through the PPT and are consistent with the simulation results. The small rod is not appeared with enough intensity here due to low statistics.

**Figure 7:**
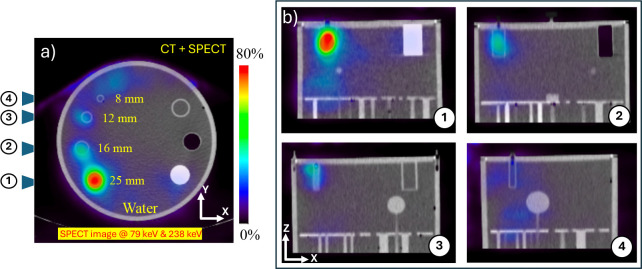
a) Fused CT and SPECT image of the clinical phantom with four cylindrical inserts filled with ^212^Pb solution (0.33 *μ*Ci/mL), acquired over 30 minutes using the StarGuide SPECT/CT system with 79 keV and 239 keV energy windows in the X–Y plane. b) Corresponding projection views of four inserts in the X–Z plane.

For comparison, we have also performed a SPECT scan of the same phantom with a StarGuide SPECT/CT scanner (GE HealthCare), a state-of-the-art commercial system comprising 12 CZT detectors arranged dodecagonally over a 2*π* angular coverage. The 79 keV and 239 keV *γ* rays from the ^212^Pb emission spectrum were used for SPECT image reconstruction. Triple energy window scatter correction was applied for both reconstructions: the energy window at 79 keV was ± 7% (high scatter window: 88 keV ± 7%; low scatter window: 67 keV ± 5%), and the energy window at 239 keV was ± 5% (high scatter window: 265 keV ± 5%; low scatter window: 215 keV ± 5%). Images were reconstructed using ordered subsets expectation maximization (OSEM), with 5 iterations and 10 subsets for the 79 keV data, and 8 iterations and 10 subsets for 239 keV data. CT-based attenuation correction was also applied. The final image combined both reconstructed images as summed images. The SPECT image obtained from a 30 minute scan is shown in Fig. 7(a–b). Notably, the larger cylindrical structures, specifically for the 25-mm diameter cylinder, are clearly visible and show comparable features to those observed in the pair production tomography images. However, the remaining three rods are less clearly resolved owing to low activity and attenuation within the collimator.

### 4.2. Experimental demonstration with small cylindrical phantom

**Figure 8:**
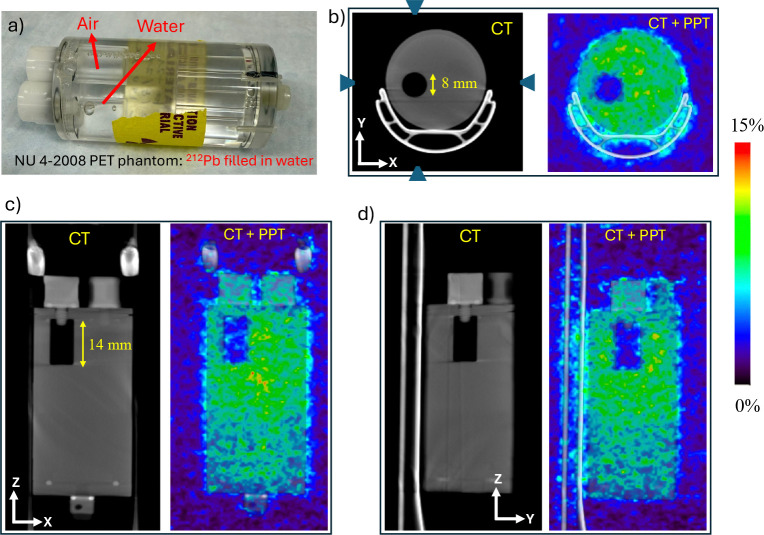
Imaging results of the NU 4–2008 small animal PET phantom filled with ^212^Pb solution (0.145 mCi in 20.9 mL), containing two internal cylindrical inserts (water and air). (a) Photograph of the phantom. (b-d) Fused CT and PPT image reconstructed from 511 keV coincidence events using the NanoScan microPET/CT system. Annihilation events are observed within the radioactive water regions, including the water filled insert and plastic material while negligible activity is seen in the air filled insert.

We performed an additional experiment using a small cylindrical phantom (NU 4–2008 PET phantom, shown in Fig. 8(a)) to demonstrate the effectiveness of time-of-flight (TOF) for PPT imaging. The phantom contains two internal cylindrical inserts (8 mm diameter and 14 mm length), one filled with non radioactive water and the other with air. The remaining volume of the phantom was uniformly filled with water containing ~5.365 MBq (0.145 mCi) of ^212^Pb dissolved in 20.9 mL of water. The phantom was first scanned using the non-TOF NanoScan microPET/CT (Mediso) system. This PET system is equipped with a 16 cm gantry bore and utilizes LYSO crystal elements with dimensions of 1.12 × 1.12 × 13 mm^3^. Combined with its proprietary reconstruction algorithm, the system achieves a PET spatial resolution of approximately 0.7 mm. The integrated CT component provides high resolution anatomical imaging with a spatial resolution of up to 30 *μ*m. The phantom was scanned for 30 minutes, and pair production image reconstruction was performed using the vendor-provided 3D iterative reconstruction algorithm with 40 iterations and 1 subset (i.e., maximum likelihood expectation maximization, not OSEM). The reconstructed CT and combined CT and PET images are shown in Fig. 8(b–d) in three orthogonal planes. As expected, negligible annihilation events are observed within the air filled cylindrical insert. In contrast, the insert containing non radioactive water shows a distribution of annihilation events arising from the passage of 2.617 MeV gamma rays through this region, which produce electron positron pairs and subsequent annihilation events. These results provide experimental validation of pair production imaging using 2.617 MeV gamma rays. However, as the NanoScan microPET system does not support time of flight capability, the reconstructed images show reduced clarity and exhibit signal within the non radioactive water region, indicating that time of flight will significantly reduce background in the reconstructed images.

**Figure 9:**
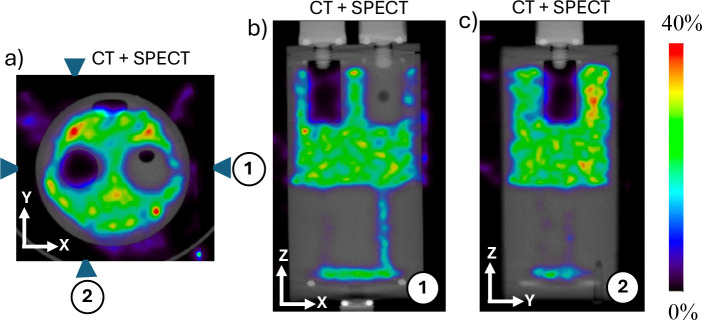
(a-c) SPECT image overlaid with CT acquired using the VETor4CT microSPECT/CT system with 79 keV and 239 keV energy windows. Two inserts, one air filled and one containing non radioactive water, are clearly distinguished from the rest volume radioactive water region.

We also performed imaging of the same phantom using a small animal SPECT scanner, the VETor4CT microSPECT/CT (MILabs), with energy windows selection of 79 keV and 239 keV, ±10% for both energy peaks. The vendor-provided automatic CT-based attenuation and triple-energy window scatter correction were applied to SPECT reconstruction. We used the HE-GP-RM that has 156 pinholes arranged as 4-pinholes clustered into one combined hole to handle high-energy photons, and provides high-sensitivity (2%) that is essential to image radionuclides like ^212^Pb. The reconstructed SPECT image, overlaid with the CT image, is shown in Fig. 9. The two internal cylindrical inserts are clearly resolved, with no detected activity within these regions, as expected.

## 5. Discussion

This study demonstrates that pair production tomography is a feasible experimental approach for imaging radionuclides that emit high energy gamma rays in the MeV range with measurable branching ratio. By using a gamma ray emission from the ^212^Pb decay chain, we obtain, to our knowledge, first experimental PPT images using 2.617 MeV gamma rays. Monte Carlo simulation results show that this signal can be localized sufficiently for PET imaging, despite the long interaction range of MeV gamma rays and the inherently low probability of pair production. The main finding of this work is that PPT expands radionuclide imaging beyond the conventional energy range of current clinical imaging without direct positron emission by radionuclide. While SPECT is highly effective for single photon imaging at sub-MeV energies, its performance degrades at higher energies owing to increased scatter and collimator penetration. By contrast, PET enables highly sensitive coincidence detection and quantitative reconstruction, but is generally restricted to radionuclides that undergo direct positron emission.

PPT addresses this limitation by transforming high energy gamma emission into a coincidence signal detectable with PET. In this context, the method goes beyond the existing imaging approaches and establishes a distinct framework for probing high energy emissions that are difficult to visualize with conventional medical scanners. This strategy is particularly relevant for current and emerging therapeutic radionuclides used in particle therapy that emit gamma rays in the MeV range, such as ^212^Pb based targeted alpha therapy. Although ^212^Pb is the administered radionuclide, therapeutic dose deposition is driven by its alpha emitting daughter products. Imaging of the parent radionuclide alone therefore may not accurately reflect the spatial distribution of these alpha emitting components. By exploiting the 2.617 MeV gamma emission from ^208^Tl, PPT provides a means to localize a therapeutically relevant daughter pathway within the decay chain.

Our simulation results clarify why such imaging is possible. Owing to its initial kinetic energy (Fig. 3(a)), the positron travels an average distance of approximately 2.2 mm before annihilating with an electron (Fig. 3(d)), a range that remains compatible with PET imaging. Although the 2.617 MeV gamma ray exhibits a long mean free path in tissue equivalent media, exponential attenuation together with isotropic geometric dispersion (1/*r*^2^) concentrates the detected signal near the source region (Fig. 3(b–f)). In water, the annihilation distribution remains localized to within ~2 mm, while the degree of localization varies systematically with material composition and density. This uncertainty is further propagated through the intrinsic PET resolution during PPT imaging. High density media such as bone preserve the signal more effectively, whereas lung tissue exhibits reduced localization owing to its lower density and extended annihilation range (Fig. 4(c) and Fig. 5(b,d)). These findings are important for interpreting PPT images in heterogeneous anatomical environments and indicate that tissue composition will influence both sensitivity and spatial resolution.

The experimental phantom studies further validate this concept. In the clinical phantom, PPT performed on a modern TOF PET system resolved the larger rods (Fig. 6) and produced image features consistent with the simulated distributions. In the preclinical small phantom, pair production imaging was observed using a non-TOF microPET system (Fig. 8), thereby confirming experimentally that the signal can be reconstructed using PPT. However, the reduced clarity and elevated background observed in the non radioactive water insert in the absence of TOF capability indicate that timing information plays a central role in practical imaging performance. These findings show that PPT can be implemented on existing PET systems, while identifying TOF capability as an important factor for enhancing localization. The comparison with SPECT is also informative. In the clinical phantom, SPECT clearly visualized the largest insert but showed reduced visibility for the smaller rods under the low activity conditions examined here (0.33 *μ*Ci/mL) (Fig. 7), owing to collimation losses and increased scatter background from high energy gamma emissions. The present results suggest that PPT may complement, rather than replace, existing SPECT based approaches for selected theranostic radionuclides with suitable high energy (MeV range) emissions.

Our PPT study at 12 kBq/mL (0.33 *μ*Ci/mL) with a 30 min acquisition lies within a clinically relevant activity range for ^212^Pb PSMA imaging. In a first in human microdose study of ^212^Pb AB001, lesion activity concentrations were estimated to be approximately 5.4 kBq/mL for a visualized lymph node metastasis using SPECT/CT, with phantom based contrast characterization extending down to 2 kBq/mL [46]. In addition, therapeutic imaging with ^212^Pb ADVC001 using SPECT/CT with energy windows at 238.6 keV and 75–91 keV, following administration of 60 MBq, demonstrated tumor uptake consistent with PSMA PET, despite the challenging imaging conditions associated with ^212^Pb, including scatter from high energy daughter emissions [45]. These comparisons indicate that PPT sensitivity at 12 kBq/mL lies within the range of activity concentrations observed in human ^212^Pb PSMA studies and may support clinically meaningful post therapy imaging. In addition, TOF total body PET systems are expected to substantially enhance sensitivity for PPT.

Regarding limitations: first, pair production remains a relatively infrequent interaction channel at 2.617 MeV, and sensitivity is therefore constrained by the branching ratio, interaction probability, scanner geometry, and counting statistics. Simulation results for a 1 cm^3^ water cube phantom indicate that approximately 97.38% of gamma rays pass through without any interaction, while 2.57% undergo Compton scattering and 0.051% result in pair production, with photoelectric absorption being negligible. Hence, approximately 2% of total interactions result in pair production. Second, reconstruction methods might be improved further for PPT, given the higher positron kinetic energies and the resulting increased positron range. Third, for in-vivo model, the daughter specific biological interpretation of the signal will depend on radionuclide kinetics and daughter redistribution, which were outside the scope of the present experiments.

These limitations outline a pathway for further development, with improvements in timing resolution, detector technology, total body PET sensitivity, event classification, and model based reconstruction expected to enhance overall performance. Scanner designs optimized for high sensitivity coincidence imaging may be particularly advantageous for PPT, especially in the low activity regime relevant to targeted alpha therapy. Beyond ^212^Pb, this conceptual framework may be extended to other radionuclides or radiation sources that produce sufficiently energetic gamma emissions. More broadly, these results suggest that the high energy components of theranostic decay spectra, often regarded primarily as a source of image degradation, can instead be harnessed for tomographic imaging.

In conclusion, we present the first experimental demonstration of pair production tomography for radionuclide imaging based on the 2.617 MeV gamma emission in the ^212^Pb decay chain. Simulation and phantom experiments studies show that pair production and subsequent annihilation events can be localized and reconstructed tomographically, with imaging performance enhanced by time of flight PET capability. These results establish PPT as a feasible approach for imaging radionuclides that emit few-MeV gamma rays and suggest a pathway for localizing therapeutically relevant daughter distributions in ^212^Pb based targeted alpha therapy. With continued advances in detector technology, timing performance, and quantitative reconstruction methods, PPT may broaden the capabilities of nuclear medicine imaging and support improved validation of emerging theranostic treatments.

## Supplementary Material

Supplement 1

## Data Availability

All the experimental data generated in this work are available from the authors upon request.

## Code availability

The GATE (Geant4) simulation code are available from the authors upon request.

## References

[1] Weber WA, Czernin J, Anderson CJ, Badawi RD, Barthel H, Bengel F, Bodei L, Buvat I, DiCarli M, Graham MM, Grimm J, Herrmann K, Kostakoglu L, Lewis JS, Mankoff DA, Peterson TE, Schelbert H, Schöder H, Siegel BA, Strauss HW. “The Future of Nuclear Medicine, Molecular Imaging,” and Theranostics. J Nucl Med. 2020 Dec; 61 (Suppl 2):263S–272S. doi: 10.2967/jnumed.120.254532. 33293447

[2] Ge J., Zhang Q., Zeng J., Gu Z., Gao M. “Radiolabeling nanomaterials for multimodality imaging: New insights into nuclear medicine and cancer diagnosis,” Biomaterials. 2020 Jan; 228:119553. 31689672 10.1016/j.biomaterials.2019.119553

[3] Vaz Sofia C., Oliveira Francisco, Herrmann Ken, Patrick Veit-Haibach, “Nuclear medicine and molecular imaging advances in the 21st century,” British Journal of Radiology, Volume 93, Issue 1110, 1 June 2020, 20200095. 32401541 10.1259/bjr.20200095 PMC10993229

[4] Rong Jian, Haider Achi, Jeppesen Troels E., Josephson Lee and Liang Steven H., “Radiochemistry for positron emission tomography,” Nat Commun 14, 3257 (2023). 10.1038/s41467-023-36377-4 37277339 PMC10241151

[5] Weber Wolfgang A., “Assessing Tumor Response to Therapy,” Journal of Nuclear Medicine May 2009, 50 (Suppl 1) 1S–10S; DOI: 10.2967/jnumed.108.057174 19380403

[6] Mahesh M, Ansari AJ, Mettler FA Jr. “Patient Exposure from Radiologic and Nuclear Medicine Procedures in the United States and Worldwide: 2009–2018,” Radiology. 2023 Apr; 307 (1):e221263. 36972184 10.1148/radiol.239006

[7] Sgouros George, Bodei Lisa, McDevitt Michael R.and Nedrow Jessie R., “Radiopharmaceutical therapy in cancer: clinical advances and challenges,” Nat. Rev. Drug Discov 19, 589–608 (2020). 32728208 10.1038/s41573-020-0073-9 PMC7390460

[8] Sartor O., de Bono J., Chi K.N., Fizazi K., Herrmann K., Rahbar K., Tagawa S.T., Nordquist L. T., Vaishampayan N., El-Haddad G., Park C.H., Beer T.M., Armour A., P´ erez-Contreras W.J., DeSilvio M., Kpamegan E., Gericke G., Messmann R.A., Morris M. J., Krause B.J., “Lutetium-177-PSMA-617 for metastatic castration-resistant prostate Cancer,” N. Engl. J. Med. 385 (2021) 1091–1103, 10.1056/NEJMoa2107322. 34161051 PMC8446332

[9] Parker C, Nilsson S, Heinrich D, “Alpha emitter radium-223 and survival in metastatic prostate cancer,” N Engl J Med. 2013; 369:213–23. 23863050 10.1056/NEJMoa1213755

[10] Cris,an G, Moldovean-Cioroianu NS, Timaru DG, Andries G, Căinap C, Chis V, “Radiopharmaceuticals for PET and SPECT Imaging: A Literature Review over the Last Decade,” Int J Mol Sci. 2022 Apr 30; 23(9):5023. doi: 10.3390/ijms23095023. 35563414 PMC9103893

[11] Arda Könik and O’Donoghue Joseph A. and Wahl Richard L. and Graham Michael M. and Van den Abbeele Annick D., “Theranostics: The Role of Quantitative Nuclear Medicine Imaging,” Seminars in Radiation Oncology, Volume 31, Issue 1, January 2021, Pages 28–36. 33246633 10.1016/j.semradonc.2020.07.003

[12] Zhang Siqi, Wang Xingkai, Gao Xin, Chen Xueyao, Li Linger, Li Guoqing, Liu Can, Miao Yuan, Wang Rui and Hu Kuan, “Radiopharmaceuticals and their applications in medicine,” Signal Transduction and Targeted Therapy volume 10, Article number: 1 (2025) 39747850 10.1038/s41392-024-02041-6 PMC11697352

[13] Burkett BJ, Bartlett DJ, McGarrah PW, Lewis AR, Johnson DR, Berberoğlu K, Pandey MK, Packard AT, Halfdanarson TR, Hruska CB, Johnson GB, Kendi AT, “A Review of Theranostics: Perspectives on Emerging Approaches and Clinical Advancements,” Radiol Imaging Cancer. 2023 Jul; 5 (4):e220157. 37477566 10.1148/rycan.220157 PMC10413300

[14] Madsen MT, “Recent advances in SPECT imaging,” J Nucl Med. 2007 Apr; 48 (4):661–73. doi: 10.2967/jnumed.106.032680. 17401106

[15] Cheng Z, Chen P, Yan J, “A review of state-of-the-art resolution improvement techniques in SPECT imaging,” EJNMMI Phys. 2025 Jan 30; 12 (1):9. doi: 10.1186/s40658-025-00724-9. 39883257 PMC11782768

[16] Peterson TE, Furenlid LR. “SPECT detectors: the Anger Camera and beyond,” Phys Med Biol. 2011 Sep 7;56(17):R145–82. doi: 10.1088/0031-9155/56/17/R01. 21828904 PMC3178269

[17] Dewaraja Yuni K., Frey Eric C., Sgouros George, Bertrand Brill A., Peter Roberson, Zanzonico Pat B., Ljungberg Michael, “Quantitative SPECT for Patient-Specific 3-Dimensional Dosimetry in Internal Radionuclide Therapy,” Journal of Nuclear Medicine Aug 2012, 53 (8) 1310–1325; DOI: 10.2967/jnumed.111.100123 22743252 PMC3465844

[18] “Michael Ljungberg, Pretorius P Hendrik, SPECT/CT: an update on technological developments and clinical applications,” British Journal of Radiology, Volume 91, Issue 1081, 1 January 2018, 20160402, 10.1259/bjr.20160402 27845567 PMC5966195

[19] Van Audenhaege K., Van Holen R., Vandenberghe S., Vanhove C., Metzler S.D. and Moore S.C. (2015), “Review of SPECT collimator selection, optimization, and fabrication for clinical and preclinical imaging,” Med. Phys., 42: 4796–4813. 10.1118/1.4927061 26233207 PMC5148182

[20] Beijst C, Elschot M, Viergever MA, de Jong HW, “A parallel-cone collimator for high-energy SPECT,” J Nucl Med. 2015 Mar;56(3):476–82. doi: 10.2967/jnumed.114.149658. 25655627

[21] Robertson A. K. H. , “Multi-isotope SPECT imaging of the 225Ac decay chain: Feasibility studies,” Phys. Med. Biol., vol. 62, no. 11, pp. 4406–4420, 2017. 28362640 10.1088/1361-6560/aa6a99

[22] Cherry SR, Jones T, Karp JS, Qi J, Moses WW, Badawi RD, “Total-Body PET: Maximizing Sensitivity to Create New Opportunities for Clinical Research and Patient Care,” J Nucl Med. 2018 Jan; 59 (1):3–12. doi: 10.2967/jnumed.116.184028. 28935835 PMC5750522

[23] Surti Suleman, “Update on Time-of-Flight PET Imaging,” J Nucl Med 2015; 56: 98–105. DOI: 10.2967/jnumed.114.145029 25525181 PMC4287223

[24] Leung EK, Abdelhafez YG, Berg E, Xie Z, Zhang X, Bayerlein R, Spencer B, Li E, Omidvari N, Selfridge A, Cherry SR, Qi J, Badawi RD. “Relating 18F-FDG image signal-to-noise ratio to time-of-flight noise-equivalent count rate in total-body PET using the uEXPLORER scanner,” Phys Med Biol. 2022 Jun 10; 67 (12). doi: 10.1088/1361-6560/ac72f1. PMC9275089 35609588

[25] Badawi Ramsey D., Shi Hongcheng, Hu Pengcheng, Chen Shuguang, Xu Tianyi, Price Patricia M., Ding Yu, Spencer Benjamin A., Nardo Lorenzo, Liu Weiping, Bao Jun, Jones Terry, Li Hongdi, Cherry Simon R., “First Human Imaging Studies with the EXPLORER Total-Body PET Scanner*,” Journal of Nuclear Medicine, 60 (3) 299–303 (2019); DOI: 10.2967/jnumed.119.226498. 30733314 PMC6424228

[26] Pasciak AS, Bourgeois AC, McKinney JM, Chang TT, Osborne DR, Acuff SN, Bradley YC. “Radioembolization and the Dynamic Role of ^90^Y PET/CT.” Front Oncol. 2014 Feb 27; 4:38. doi: 10.3389/fonc.2014.00038. 24579065 PMC3936249

[27] D’Arienzo M, “Emission of β+ Particles Via Internal Pair Production in the 0+ – 0+ Transition of 90Zr: Historical Background and Current Applications in Nuclear Medicine Imaging. Atoms. 2013; 1(1): 2–12. 10.3390/atoms1010002

[28] Kao YH, Tan EH, Lim KY, Ng CE, Goh SW. “Yttrium-90 internal pair production imaging using first generation PET/CT provides high-resolution images for qualitative diagnostic purposes.” Br J Radiol. 2012 Jul; 85(1015): 1018–9. doi: 10.1259/bjr/33524085. 21976634 PMC3474067

[29] Gates VL, Esmail AA, Marshall K, Spies S, Salem R. “Internal pair production of ^90^Y permits hepatic localization of microspheres using routine PET: proof of concept.” J Nucl Med. 2011 Jan; 52(1): 72–6. doi: 10.2967/jnumed.110.080986. 21149493

[30] Lyu Qihui, Neph Ryan, Sheng Ke, “Tomographic detection of photon pairs produced from high-energy X-rays for the monitoring of radiotherapy dosing.” Nature Biomed. Eng. 2023 Mar;7(3):323–334. doi: 10.1038/s41551-022-00953-8. 36280738 PMC10038801

[31] Roghiye Bodaghi Hosseinabadi Hossein Rajabi, “Real-time dosimetry in lung cancer radiotherapy using PET imaging of positrons induced by gold nanoparticles,” Journal of Radiation Research and Applied Sciences Volume 18, Issue 2, June 2025, 101361.

[32] Berckmans Y, Kleynhans J, Van Mechelen S, Goffin K, Baete K, Koole M, Coosemans A, Cocolios TE, Deroose CM, Seimbille Y, Cleeren F, “Lead radionuclides for theranostic applications in nuclear medicine: from atom to bedside,” Theranostics 2026;16(6):2887–2917. doi:10.7150/thno.126086. 41510167 PMC12775822

[33] Delpassand ES, Tworowska I, Esfandiari R, Torgue J, Hurt J, Shafie A, Núñez R, “Targeted *α*-Emitter Therapy with ^212^Pb-DOTAMTATE for the Treatment of Metastatic SSTR-Expressing Neuroendocrine Tumors: First-in-Humans Dose-Escalation Clinical Trial,” J Nucl Med. 2022 Sep;63(9):1326–1333. doi: 10.2967/jnumed.121.263230. 34992153 PMC9454455

[34] Strosberg Jonathan R., Naqvi Shagufta, Allen Lee Cohn, Delpassand Ebrahim S, Volker Jean Wagner, Izabela Tworowska, Julien Torgue, Rachel Woloski, Allison Manuel, and Maluccio Mary Alice, “Safety, tolerability and efficacy of 212PbDOTAMTATE as a targeted alpha therapy for subjects with unresectable or metastatic somatostatin receptor-expressing gastroenteropancreatic neuroendocrine tumors (SSTR+ GEP-NETs): A phase 2 study,” J Clin Oncol 42, 4020 (2024).

[35] Ramonaheng Keamogetswe, Qebetu Milani, Banda Kaluzi, Goorhoo Pryaska, Legodi Khomotso, Mdanda Sipho, Sibiya Sandile, Mzizi Yonwaba, Ndlovu Honest, Kabunda Joseph, Yang Mengdie, Shi Kuangyu, Sathekge Mike, “Advances in Dosimetry and Imaging for 203Pb and 212Pb Radiotheranostics,” Seminars in Nuclear Medicine, Volume 55, Issue 6, 2025, Pages 1011–1031, ISSN 0001–2998, 10.1053/j.semnuclmed.2025.09.006. 41062349

[36] Juzeniene A, Stenberg VY, Bruland ØS, Revheim M-E and Larsen RH, “Dual targeting with 224Ra/212Pb-conjugates for targeted alpha therapy of disseminated cancers: A conceptual approach,” Front. Med. 9:1051825 (2023). doi: 10.3389/fmed.2022.1051825 PMC9887039 36733936

[37] Kästner D., Hartmann H., Freudenberg R. , “Gamma camera imaging characteristics of ^203/212^Pb as a theragnostic pair for targeted alpha therapy: a feasibility study,”. EJNMMI Phys 12, 50 (2025). 10.1186/s40658-025-00763-2 40419836 PMC12106261

[38] Kvassheim M, Revheim MR, Stokke C., “Quantitative SPECT/CT imaging of lead-212: a phantom study,” EJNMMI Phys. 2022 Aug 4; 9(1):52. doi: 10.1186/s40658–022-00481-z. Erratum in: EJNMMI Phys. 2022 Oct 10;9(1):71. doi: 10.1186/s40658-022-00499-3. 35925521 PMC9352840

[39] NNDC Homepage, [accessed on February 2025], https://www.nndc.bnl.gov.

[40] Karp Joel S., Surti Suleman, Daube-Witherspoon Margaret E.and Gerd Muehllehner, “Benefit of Time-of-Flight in PET: Experimental and Clinical Results,” Journal of Nuclear Medicine March 2008, 49 (3) 462–470; DOI: 10.2967/jnumed.107.044834. PMC2639717 18287269

[41] Lois Cristina, Jakoby Bjoern W., Long Misty J., Hubner Karl F., Barker David W., Casey Michael E., Conti Maurizio, Panin Vladimir Y., Kadrmas Dan J. and Townsend David W., “An Assessment of the Impact of Incorporating Time-of-Flight Information into Clinical PET/CT Imaging,” Journal of Nuclear Medicine February 2010, 51 (2) 237–245; DOI: 10.2967/jnumed.109.068098. PMC2818518 20080882

[42] Akamatsu Go, Ishikawa Kaori, Mitsumoto Katsuhiko, Taniguchi Takafumi, Ohya Nobuyoshi, Baba Shingo, Abe Koichiro and Sasaki Masayuki, “Improvement in PET/CT Image Quality with a Combination of Point-Spread Function and Time-of-Flight in Relation to Reconstruction Parameters,” Journal of Nuclear Medicine November 2012, 53 (11) 1716–1722. 22952340 10.2967/jnumed.112.103861

[43] Chaudhari Akshay S., Mittra Erik, Davidzon Guido A., Gulaka Praveen, Gandhi Harsh, Brown Adam, Zhang Tao, Srinivas Shyam, Gong Enhao, Zaharchuk Greg and Jadvar Hossein, “Low-count whole-body PET with deep learning in a multicenter and externally validated study.” npj Digit. Med. 4, 127 (2021). 34426629 10.1038/s41746-021-00497-2 PMC8382711

[44] Sangeeta Ray Banerjee Il Minn, Kumar Vivek, Josefsson Anders, Lisok Ala, Brummet Mary, Chen Jian, Kiess Ana P., Baidoo Kwamena, Brayton Cory, Mease Ronnie C., Brechbiel Martin, Sgouros George, Hobbs Robert F., Pomper Martin G., “Preclinical Evaluation of ^203/212^Pb-Labeled Low-Molecular-Weight Compounds for Targeted Radiopharmaceutical Therapy of Prostate Cancer,” Journal of Nuclear Medicine Jan 2020, 61 (1) 80–88; DOI: 10.2967/jnumed.119.229393. PMC6954458 31253744

[45] Griffiths Matthew R., Pattison David A., Latter Melissa, Kuan Kevin, Taylor Stephen, Tieu William, Kryza Thomas, Meyrick Danielle, Boon Quan Lee Aaron Hansen, Rose Stephen E., Puttick Simon G., “First-in-Human 212Pb-PSMA–Targeted *α*-Therapy SPECT/CT Imaging in a Patient with Metastatic Castration-Resistant Prostate Cancer,” Journal of Nuclear Medicine Feb 2024, jnumed.123.267189; DOI: 10.2967/jnumed.123.267189 PMC10995529 38423783

[46] Berner K, Hernes E, Kvassheim M, Revheim ME, Bastiansen J, Selboe S, Bakken CL, Grønningsæter SR, Bruland ØS, Larsen RH, Bouzelmat L, Jardine VL, Stokke C. “First-in-Human Phase 0 Study of AB001, a Prostate-Specific Membrane Antigen-Targeted 212Pb Radioligand, in Patients with Metastatic Castration-Resistant Prostate Cancer.” J Nucl Med. 2025 May 1;66(5):732–738. doi: 10.2967/jnumed.124.269299. 40081958 PMC12051763

[47] Moskal Paweł , “Positronium image of the human brain in vivo.” Sci. Adv.10, eadp2840(2024).DOI:10.1126/sciadv.adp2840 39270027 PMC11397496

[48] Conti M., Eriksson L. Physics of pure and non-pure positron emitters for PET: a review and a discussion. EJNMMI Phys 3, 8 (2016). 10.1186/s40658-016-0144-5 27271304 PMC4894854

[49] Abuelhia E., Kacperski K. and Spyrou N.M. “Three-photon annihilation in PET: 2D imaging experiments.” J Radioanal Nucl Chem 271, 489–495 (2007). 10.1007/s10967-007-0235-9

[50] Berger M.J., Hubbell J.H., Seltzer S.M., Chang J., Coursey J.S., Sukumar R., Zucker D.S., and Olsen K., “XCOM: Photon Cross Sections Database Share: NIST Standard Reference Database 8 (XGAM),” Last Update to Data Content: November 2010 | NBSIR 87–3597 | DOI: 10.18434/T48G6X.

