# Supplementary material for: Pair production tomography enables imaging of MeV-scale gamma-emitting theranostic radionuclides
Source: Res Sq. 2026 May 4:rs.3.rs-9349692. Preprint. [Version 1] doi: 10.21203/rs.3.rs-9349692/v1 (PMC13174768; doi:10.21203/rs.3.rs-9349692/v1)
Supplement: Supplement 1 [file NIHPPRS9349692v1-supplement-1.pdf]

1. Supplemental Data

1.1. GATE simulation material data

Below are the material data used for the GATE simulations in the .db file.

```
Water: d=1.00 g/cm3; n=2 ; state=liquid
        +el: name=Hydrogen   ; n=2
        +el: name=Oxygen     ; n=1

RibBone: d=1.92 g/cm3 ; n=11
        +el: name=Hydrogen   ; f=0.034
        +el: name=Carbon     ; f=0.155
        +el: name=Nitrogen   ; f=0.042
        +el: name=Oxygen     ; f=0.435
        +el: name=Sodium     ; f=0.001
        +el: name=Magnesium  ; f=0.002
        +el: name=Phosphor   ; f=0.103
        +el: name=Sulfur     ; f=0.003
        +el: name=Calcium    ; f=0.225

Lung:  d=0.26 g/cm3 ; n=9
        +el: name=Hydrogen   ; f=0.103
        +el: name=Carbon     ; f=0.105
        +el: name=Nitrogen   ; f=0.031
        +el: name=Oxygen     ; f=0.749
        +el: name=Sodium     ; f=0.002
        +el: name=Phosphor   ; f=0.002
        +el: name=Sulfur     ; f=0.003
        +el: name=Chlorine   ; f=0.003
        +el: name=Potassium  ; f=0.002

Adipose: d=0.92 g/cm3 ; n=11
        +el: name=Hydrogen  ; f=0.120
        +el: name=Carbon    ; f=0.640
        +el: name=Nitrogen  ; f=0.008
        +el: name=Oxygen    ; f=0.229
        +el: name=Phosphor  ; f=0.002
        +el: name=Calcium   ; f=0.001
```

1.2. Cube simulation extended methods and interpretation

All GATE cube simulations were conducted with the emstandard_opt3 physics list. List-mode tracking simulations in the 10 cm × 10 cm × 10 cm phantom for e^+ KE and range quantification were conducted without voxelization or range cuts. This simulation is computationally expensive, so to assess the source profile in the larger 20 cm × 20 cm × 20 cm phantom in several materials, the GATE Production and Stopping actor was used with 0.1 mm range cuts and 0.5 mm voxelization.

In Fig. 4a, the end-point e^+ energy of 1.58 MeV is approximately 35 keV lower than $2.617\,\mathrm{MeV} - 2m_0c^2 = 1.595\,\mathrm{MeV}$. This deficit can likely be attributed to bremsstrahlung escape from the simulated phantom, as the e^+ KE was computed by summation of the energy deposition processes from each unique pair production history.

Because of the γ-ray range, larger cube phantoms will increase the maximum pair production interaction position such that larger phantoms provide wider profiles. The 20-cm cube used in the main results represents an approximate patient thickness. 1D slice profiles (Fig. 4b–c) and 2D XZ images (Fig. 4e–f) were obtained by selecting the center slice. In other words, for a voxel size of 0.5 mm, x_p were selected where $y_p, z_p \leq 0.5\,\mathrm{mm}$ (or only y_p for 2D XZ). However, conventions may differ for representation of the FWHM of a 3D distribution. Larger slice thickness, including a full x-coordinate projection, will convey a larger estimate of the point-spread than a slice (Supp. Fig. S1).

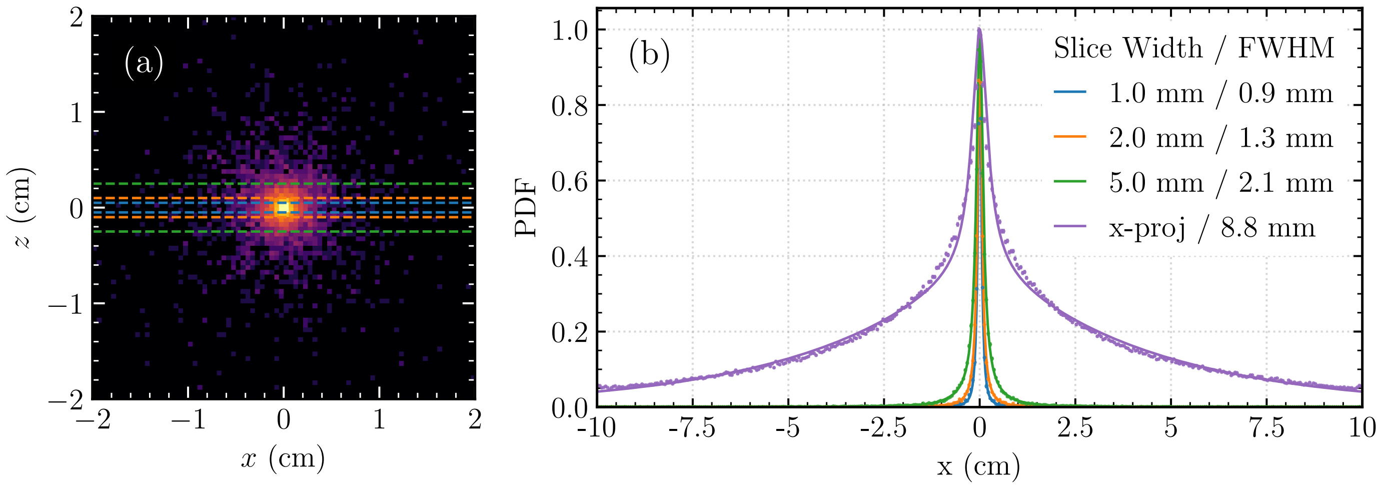

Figure S1: Characterization of a the e^+ production 3D kernel with varied 1D slice thicknesses, compared to a full x-projection. Dashed lines in (a) show the boundaries of z-slices, and similar in y, in which x-coordinates were collected to produce 1D profiles in (b).

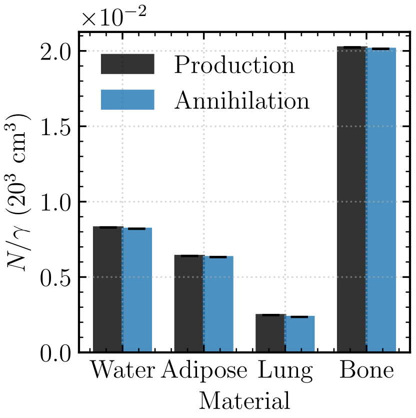

Figure S2: In the full 20 cm × 20 cm × 20 cm cube simulation, nearly all produced positrons also annihilate in-phantom.
